# Micro-Structure Changes Caused by Thermal Evolution in Chalcogenide Ge_x_As_y_Se_1−x−y_ Thin Films by In Situ Measurements

**DOI:** 10.3390/ma14102572

**Published:** 2021-05-15

**Authors:** Xueqiong Su, Yong Pan, Dongwen Gao, Shufeng Li, Jin Wang, Rongping Wang, Li Wang

**Affiliations:** 1 College of Applied Sciences, Beijing University of Technology, Beijing 100124, China; nysxq@bjut.edu.cn (X.S.); panyong_paper@126.com (Y.P.); gaodongwen@emails.bjut.edu.cn (D.G.); lishufeng@emails.bjut.edu.cn (S.L.); wangjin321@emails.bjut.edu.cn (J.W.); 2 Centre for Ultra-High Bandwidth Devices for Optical Systems, Laser Physics Centre, Australian National University, ACT, 2600 Canberra, Australia; wangrongping@nbu.edu.cn

**Keywords:** chalcogenide glasses, Ge_x_As_y_Se_1−x−y_, conduction mechanisms, electrical conductivity

## Abstract

To understand the effects of thermal annealing on the structure of Ge_x_As_y_Se_1−x−y_ thin films, the thermal evolution of these films was measured by the in situ X-ray diffraction (XRD) at different temperature (773 K or 1073 K) in a vacuum (10^−1^ Pa) environment. The entire process of crystallization can be observed by using in situ XRD, which is from the appearance of a crystal structure to melting liquid-state and ultimately to the disappearance of the amorphous structure. In the crystallized process, the corresponding state-transition temperatures T_x_ (the onset crystallization temperature), T_l_ (the transition temperature from glassy-state to liquid-state), T_p_ (peak crystallization temperature) are linear with MCN (Mean Coordination Number). In order to obtain information about changes in the amorphous structural origin of the anneal-induced material, the samples were analyzed by in situ Raman spectroscopy. Analysis of the results through decomposing the Raman spectra into different structural units showed that the Ge−Ge, As−As, or Se−Se homopolar bonds as the nonequilibrium minority carriers could be found in films. It suggests that the formation of these bonds cannot be completely suppressed in any case, as one falls and another rises.

## 1. Introduction

Chalcogenide glasses are the networks formed usually along with Si, P, Ge, As, Ge, or Sb containing one or more of the chalcogen elements, such as sulfur, selenium, and tellurium from group VI of the periodic table. Chalcogenide glasses, an important photonics material, are applied to mid-infrared waveguide devices, supercontinuum optical fiber, optical modulators, and non-volatile memory [1] due to their unique properties [2,3,4]. Under certain voltage, illumination, or heating conditions, it is necessary to study the threshold switching mechanism of chalcogenide glasses to obtain the changes of crystal texture, optical refractive index, or resistance [5,6,7]. Although it is generally believed that threshold switching is an important electronic process, its specific physical mechanism is still suspected [8,9,10], which is also considered as a kind of trapped carrier transport, namely small polaron transition [11,12,13,14] or thermally induced Poole Frenkel pf conduction [15,16]. The systematic introduction of the threshold switch is the transport mechanism of the local state in an amorphous semiconductor conceptually, especially in a nonequilibrium state [17,18,19]. Zhang et al. [20] studied the conductivity of two-dimensional chalcogenide material by using density functional theory and experimental heating methods. The results show that the conductivity of the two-dimensional material can reach 192 cm^2^ V^−1^ s^−1^. They believed that it is a promising material for nano electronic devices. In addition, the structural changes of amorphous chalcogenide films are still the basis of their applications and mechanisms. The relationship between the optical refractive index of Ge_x_As_y_Se_1−x−y_ chalcogenide glass film under illumination and the glass transition temperature and crystal state under heating was studied, and the most suitable and stable optical waveguide material was found. The effect of light on the structure and composition of amorphous sulfur system materials was studied by Zhang et al. [21]. The results show that the best glass composition for waveguide manufacturing is Ge_15_ series chalcogenide materials. We know that for the chalcogenide material, the main properties of the material will be changed as the different compositions of elements. Generally speaking, the increase in each element, such as Ge, will change the application direction of materials. The reason is different elements bonding in different ways, which eventually leads to the different microstructure. However, there are still problems that need to be solved further. The research method of structural change has a profound influence on the application and main properties of materials. Although the best material selection of optical waveguide can be obtained by illumination, it is not a favorable research method for chalcogenide materials. The dependence of elements in chalcogenide materials on temperature cannot be underestimated, but there is a lack of such a systematic research method at present.

In this research, it is more conveniently observed under high-resistance chalcogenide glass thin film, exploring the threshold conversion rule of crystalline and electrical properties of Ge_x_As_y_Se_1−x−y_ chalcogenide glass thin-film MCN (Mean Coordination Number) under heating. Before this, the properties of Ge_x_As_y_Se_1−x−y_ have been systematically characterized [22]. Here, the in situ heating method is used in the testing of XRD and Raman. The purpose of our study is to find out the effect of temperature on the microstructure of materials accurately by this method so as to guide our next research on the application of chalcogenide materials. Therefore, this study has reference value for the application of chalcogenide materials.

## 2. Materials and Methods

### 2.1. Source Material

The source material Ge_x_As_y_Se_1−x−y_ (Aldrich, Shanghai, China) was composed of Ge (99.99%, 4N), As (99.99%, 4N), Se (99.99%, 4N). The target was prepared by the traditional melt–quenching technique. The different compositions of Ge_x_As_y_Se_1−x−y_ bulk glasses ranged from 5 < x < 33 and 5 < y < 35. 

### 2.2. Thermal Evaporation

One hundred millimeter-diameter Silicon wafers (Aldrich, Shanghai, China) were placed at a distance of 40 cm from the baffled box evaporation source that was set in a sample holder rotated in planetary motion. Before it was deposited, the pressure of the chamber was evacuated to 10^−6^ Torr, and then an ion gun irradiated the substrates by a 50 eV, 1A Ar+ beam for 3 min aiming to enhance the impurities and surface cleanliness. The films with a thickness of 1 µm were deposited at a rate of 2–5 Å/s. For the various experiments, they were then cut into small film pieces with the size of 2 × 2 cm^2^.

### 2.3. In Situ Measurements

The samples were heated on a Pt hot plate and subjected to a real-time XRD test to monitor the phase transition of the thin film under a continuous heating environment. The temperature heated from room temperature to 900 °C with a step of 100 °C, then cooled to room temperature and was monitored for phase reversibility in this process. The resistance of Ge_x_As_y_Se_1−x−y_ chalcogenide glass film was measured by the four-probe method and a real-time variable temperature heating resistance test due to the higher sheet resistance. The sample area was evacuated to 10^−2^ Pa to avoid the influence of airflow on the resistance change during the testing for accurate resistance changes.

### 2.4. Characterization

According to the energy dispersive X-ray analysis (EDX) installed under a scanning electron microscope (JEOL Ltd., Tokyo, Japan) using commercial Ge_33_As_12_Se_55_ as the reference standard, the chemical compositions of the bulk glass and the films were accessible to be analyzed. The amorphicity of the bulk glass and films was examined by X-ray diffraction (XRD) using a traditional X-ray diffractometer (Bruker, Karlsruhe, Germany) in a 2θ scan. The information on bonds vibration and micro-structure was revealed by Raman spectra (BWTEK MiniRam II, Newark, NJ, USA). The electrical resistance was tested by the Hall-effect (Tektronix, Inc., Beaverton, OR, USA). The film surface morphology was tested by AFM (Atomic Force Microscope) using Veeco (Multimode, non-contact measurement, 256 × 256, Bruker, Santa Barbara, CA, USA) instrument.

## 3. Results and Discussion

### 3.1. Structural Properties

**Figure 1:**
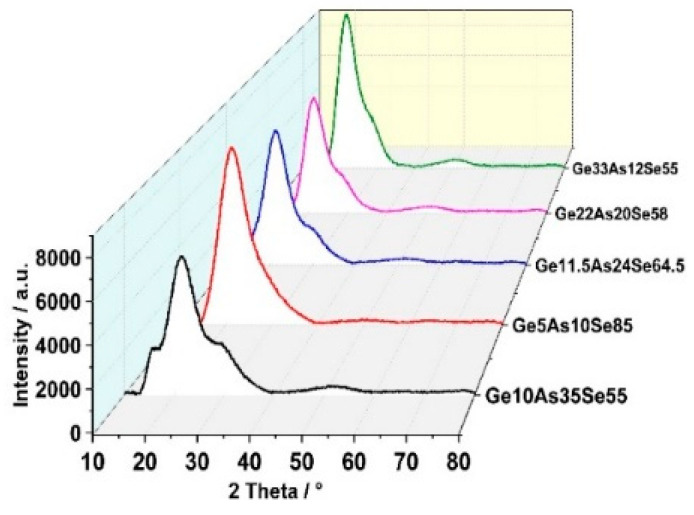
X-ray diffraction patterns of Ge_x_As_y_Se_1−x−y_ thin films measured at room temperature.

To study the effects of thermal annealing on the structure of Ge_x_As_y_Se_1−x−y_ thin films, the thermal evolution of these films was measured by in situ X-ray diffraction (XRD) in the temperature range from 773 K to 1073 K in a vacuum (1–10 Pa). Figure 1 shows the X-ray diffraction patterns of Ge_x_As_y_Se_1−x−y_ thin films without thermal annealing. A long-range structural disorder characteristic of the amorphous network in chalcogenide glasses films was confirmed by the asymmetric and broad peaks. These curves indicated, regardless of the difference in chemical composition, the amorphous nature of all initially prepared films.

**Figure 2:**
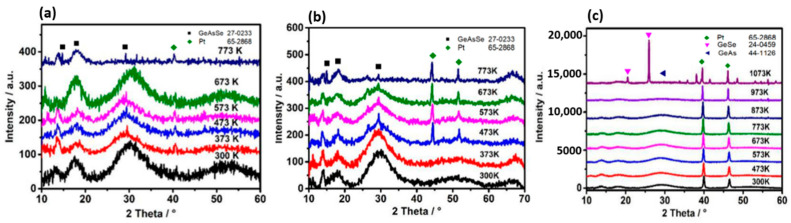
The in situ x-ray diffraction patterns of Ge_x_As_y_Se_1−x−y_ thin films were measured by the X-ray diffraction (XRD) up to temperature (773 K or 1073 K) in vacuum (10^−1^ Pa) measured at room-temperature. (**a**) Ge_20_As_10_Se_70_, (**b**) Ge_17.5_As_11_Se_72.5_ and (**c**) Ge_33_As_12_Se_55_.

**Figure 3:**
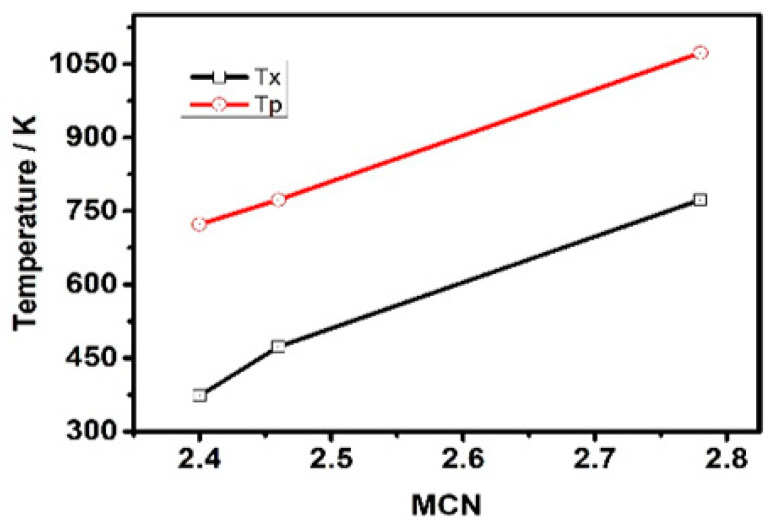
Two transition temperatures (the onset crystallization temperature T_x_ and peak crystallization temperature T_p_) as a function of MCN (Mean Coordination Number).

Figure 2 shows the in situ XRD curves of the Ge_x_As_y_Se_1−x−y_ thin films under different annealing temperatures: (a) Ge_10_As_20_Se_70_, (b) Ge_17.5_As_11_Se_72.5_ and (c) Ge_33_As_12_Se_55_. The reason why we choose these three samples to measure by in situ XRD was that they had different resistance changing trends, as mentioned above. The second reason was that MCN values of these three films were distributed in the low, middle, and high values of 2.4~2.78, respectively. For the glassy system Ge_a_As_b_Se_c_ (a + b + c = 1), the value of MCN is given by: MCN = 4a + 3b + 2c. Each XRD curve in Figure 2 shows the cubic Pt phase with (111) diffraction peak at 39.97°, (200) diffraction peak at 46.52°, and (220) diffraction peak at 68.16°. The above peaks can be assigned to the underlying Pt heating wafer because the XRD is an in situ heating test method. Therefore, when heating the substrate, there was a Pt heating source under the substrate, and then it was heated while testing. Therefore, the peak position of Pt was detected at that moment. Initially, broad peaks at 29.5°,17.9° and 13.85° were observed, meaning the existence of the amorphous glass phase under 300K in three samples. Then the higher annealing temperature developed preferential growth of identified crystal phases and a significant reduction in the broad peak intensity. The sharp peaks at 29.34° and 14.95° of Ge_17.5_As_11_Se_72.5_ and Ge_10_As_20_Se_70_ samples corresponded to the crystal GeAsSe phase (Jade PDF number: 27-0233). In the case of the Ge_33_As_12_Se_55_ sample, the (201) peaks at 26.52°, (110) peaks at 20.84° corresponded to the Hexagonal GeSe phase (Jade PDF number: 24-0459). And the (310) diffraction peak at 31.76° corresponded to the Monoclinic GeAs phase (Jade PDF number: 44-1126). Further increments, up to 773 K or 1073 K, resulted in the vanishing of the broad, amorphous peak at 29.5°. It was found that the position of lattice peaks was related to the chemical composition content. The most convincing evidence was that the Ge_33_As_12_Se_55_ sample showed different peak positions for rich-Ge content. According to [23], the glass transition temperature T_g_ of chalcogenide glass depends on the Mean Coordination Number (MCN, defined as the sum of the respective elemental concentrations times their covalent coordination number) and thus depends on the rigidity of the vitreous network. Clearly, from glassy state to crystal state, there were two transition points by in situ XRD whose corresponding transition temperatures were onset crystallization temperature T_x_ and peak temperature T_p_. When continuing to heat the glass over T_g_, crystallization occurred at Tx, and the best crystal peak existed at T_p_. Both T_x_ and T_p_ also depended on the MCN and the rigidity of the vitreous network, shown in Figure 3. It is worth mentioning that a completely amorphous phase suddenly appeared between T_x_ and T_p_ in the Ge_10_As_20_Se_70_ film sample with the lowest MCN value. The lower MCN, the lower transition temperature was confirmed, including T_x_, T_p_, and T_l_. Therefore, the amorphous phase reappeared at the temperature T_l_, which was the transition temperature from glassy-state to liquid-state. Essentially, for the chalcogenide glass films, the electron distribution of the outer layer of the atom and the network structure of molecules were major determinants of resistance and crystal phase with the increase in annealing temperature. Using in situ XRD, the entire process of crystallization could be observed, which was from the appearance of the crystal structure to melting liquid-state and ultimately to the disappearance of the amorphous structure. In the crystallization process, the corresponding state-transition temperatures T_x_, T_l_, T_p_ were linear with MCN. The influence of composition on different lattice structures could also be judged by the peak position. Based on the above results, it is accessible to expand the glass phase region and increase the initial crystallization temperature by introducing targeted protective atmospheres or doping inhibitors.

### 3.2. In Situ Raman

**Figure 4:**
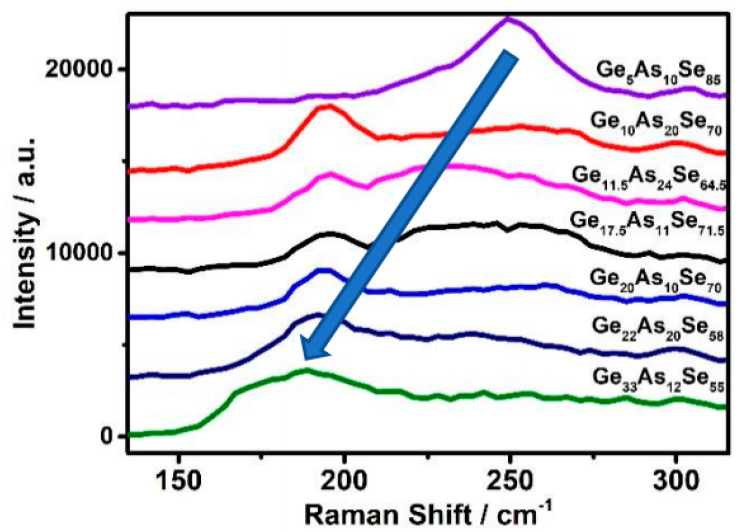
The Raman patterns of Ge_x_As_y_Se_1−x−y_ thin films measured at room temperature.

The samples were analyzed by Raman spectroscopy. The broad peaks appeared in the Raman spectra between 150 cm^−1^ and 330 cm^−1^ for the amorphous nature of the glasses and the proximity of the elements in the periodic table. Figure 4 depicts the Raman spectra for the initial GeAsSe deposited with increasing MCN by changing the contents ratio and the results of decomposing the spectra into individual peaks. With the MCN and germanium content increasing, the position of the broad peak occurred in a blue shift. The excited light of Raman was 785 nm.

**Figure 5:**
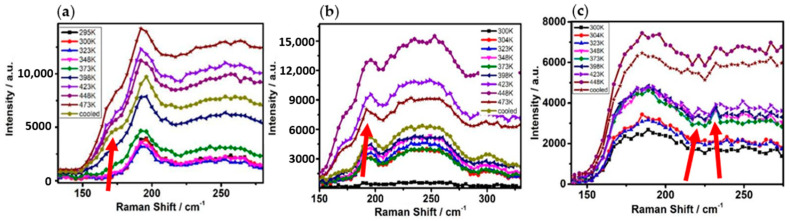
The in situ Raman patterns of Ge_x_As_y_Se_1−x−y_ thin films tested at a temperature of 473K. (**a**) Ge_20_As_10_Se_70_, (**b**) Ge_17.5_As_11_Se_72.5_ and (**c**) Ge_33_As_12_Se_55_.

Figure 5 shows the in situ Raman patterns of Ge_x_As_y_Se_1−x−y_ thin films heated to temperature (473K), (a) Ge_20_As_10_Se_70_, (b) Ge_17.5_As_11_Se_72.5_ and (c) Ge_33_As_12_Se_55_. Choosing this temperature can avoid annealing temperatures higher than that of the glass transition temperature T_g_. With the increase in annealing temperature, the Raman signal intensity increased greatly for phononic vibration amplitude.

The red arrow indicates the location of the new peak. When the temperature rose to 423 K, the red scissors showed that the new peak appeared near 173 cm^−1^ in Figure 5a,b, 231 cm^−1^ and 221 cm^−1^ in Figure 5c. The new Raman modes of Ge−Ge homopolar bond were observed in Ge_20_As_10_Se_70_ and Ge_17.5_As_11_Se_72.5_ samples, A1(ν2) modes of As_4_Se_3_ cage-like molecules in Ge_33_As_12_Se_55_ sample.

**Figure 6:**
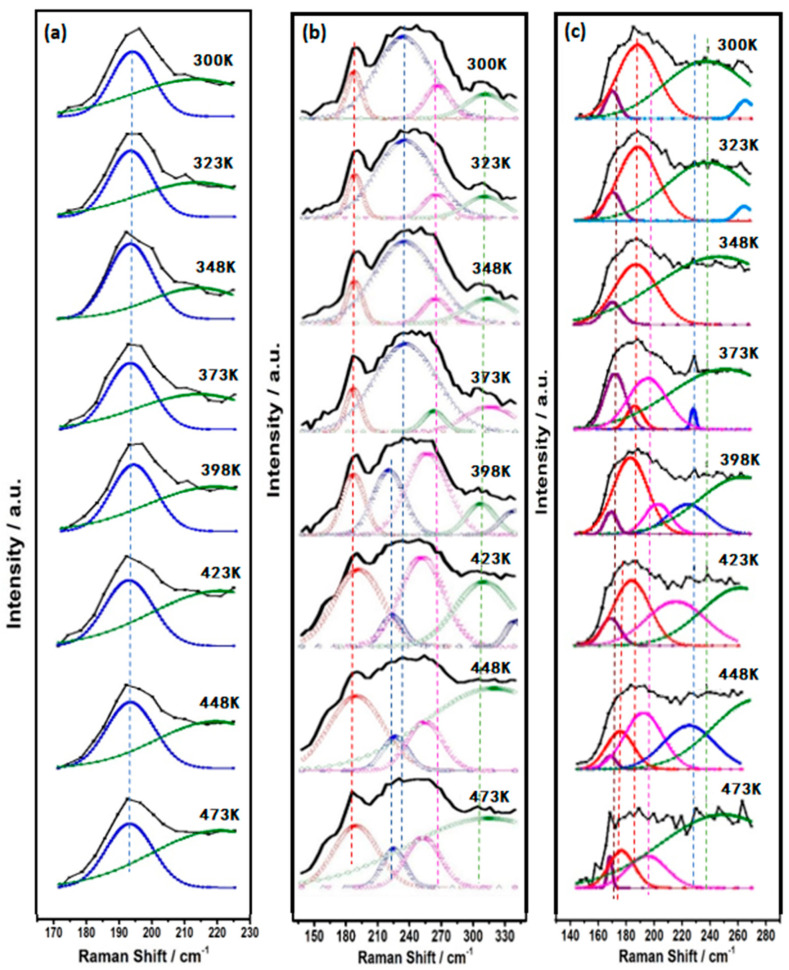
The set of in situ Raman spectra of Ge_x_As_y_Se_1−x−y_ thin films up to temperature (473 K), decomposing the spectra into individual peaks. (**a**) Ge_20_As_10_Se_70_ (**b**) Ge_17.5_As_11_Se_72.5_ (**c**) Ge_33_As_12_Se_55_ and Ge_20_As_10_Se_70_.

Because the wide Raman peak corresponded to the overlapping individual vibration peak, the Raman peak was decomposed into several characteristic vibration peaks by computer simulation. Each characteristic vibration peak represented a single characteristic of each vibration that can be directly recognized. Figure 6 shows that the Ge−As−Se glass network consists of basic structural units, specifically GeSe_4/2_ tetrahedron, AsSe_3/2_ pyramid, and Ge−Ge or Se−Se homopolar bonds. Raman peaks drifted with the increase in temperature. Some of them had a blue shift; others had a red shift. The reason for this phenomenon is that the change in bonding energy leads to the change in microstructure. The peaks at 195 cm^−1^ and 213 cm^−1^ can be attributed to Corner-sharing (CS) and edge-sharing (ES) GeSe_4/2_ tetrahedral units [24,25]. The bands with their maximum at 230 cm^−1^ and 268 cm^−1^ were mainly connected to the AsSe_3/2_ pyramidal unit [26]. In addition, ethane-like Ge–Ge bond vibration modes could be observed at ~300cm^−1^ and ~175 cm^−1^ in Se-poor glasses [27,28]. The vibrational modes of Se chains or rings had Raman peaks at 250 cm^−1^ [24]. The As–As vibration modes had Raman peaks at 247 cm^−1^ [26,29,30,31,32,33].

**Figure 7:**
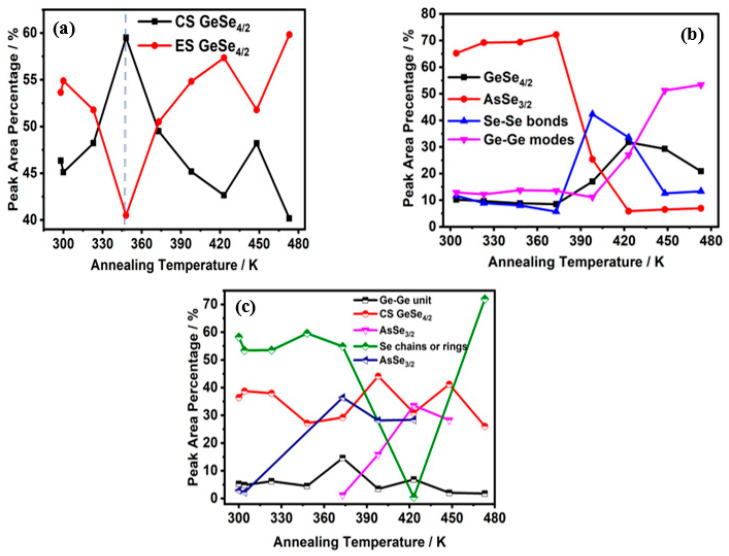
The relative ratio of the area of each decomposed peak to the entire integrated area of Raman spectra, (**a**) Ge_20_As_10_Se_70_ (**b**) Ge_17.5_As_11_Se_72.5,_ (**c**) Ge_33_As_12_Se_55._

To see the evolution of the bonds with increasing annealing temperature clearly, Figure 7 presents the relative ratio of the area of each decomposed peak to the entire integrated area of Raman spectra as a function of annealing temperature. Obviously, GeSe_4/2_ bonds existed in all cases, and the content was not less than 30%. The percentage of new peaks mentioned in Figure 5 was too small compared with the original area, so they did not appear in Figure 6 and Figure 7. For Ge_20_As_10_Se_70_ film, the GeSe_4/2_ Corner-sharing (CS) mode and GeSe_4/2_ edge-sharing (ES) mode transformed each other near the glass transition temperature. For Ge_17.5_As_11_Se_72.5_ film, the contribution of Ge–Se increased while that of As–Se decreased. Specifically, the content of Ge−Ge bonds changed from 13% up to about 53%. At the same time, the content of the Se−Se bond decreased from 42% to 13%. For the Ge_33_As_12_Se_55_ film, the contribution of Se–Se and As–Se bonds were converted to each other. As one fell, another rose. Homopolar Ge−Ge and Se−Se bonds all existed in both of rich-Se film Ge_17.5_As_11_Se_72.5_ or rich-Ge film Ge_33_As_12_Se_55_. With the increase in temperature, chemical bonds affected or inhibited each other near the glass transition temperature.

In our previous work, the refractive index and the optical bandgap were almost constant under long-time thermal annealing or irradiation by sub-bandgap light [34,35,36]. Generally, Ge_x_As_y_Se_1−x−y_ thin films had high resistance values ranging from 10^12^ to 10^13^ Ω. The reason can be explained by the fact that a large concentration of donor and acceptor states that exist near the Fermi level tend to be localized in the energy gap. For the Ge−As−Se high resistivity structure system, under unbalanced conditions, the number of nonequilibrium minority carriers changes significantly, resulting in a strong change in electrical performance. So the changes of the Ge−Ge and Se−Se homopolar bonds are more concerned.

**Figure 8:**
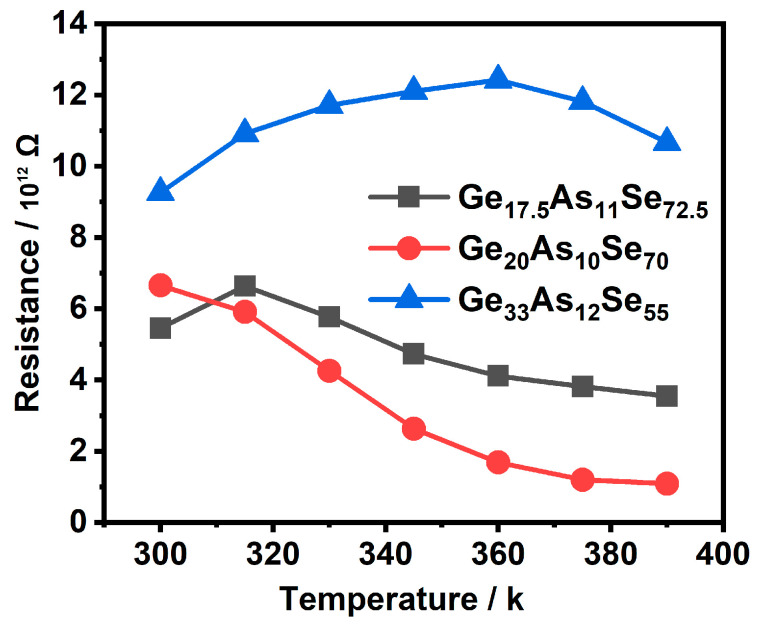
The electrical resistance change in Ge_x_As_y_Se_1−x−y_ thin films with annealing temperature.

The resistance decreased with the increase in annealing temperature in Ge_20_As_10_Se_70_ film, as shown in Figure 8. With the increase in annealing temperature, the resistance increased first and then decreased in rich-Se Ge_17.5_As_11_Se_72.5_ film and rich-Ge Ge_33_As_12_Se_55_ film. For rich-Se and rich-Ge films, the nonequilibrium minority carriers were Ge−Ge or As−As bonds. Specifically, the number of Ge−Ge bonds of Ge_20_As_10_Se_70_ was relatively small, so the electrical properties changed greatly.

**Figure 9:**
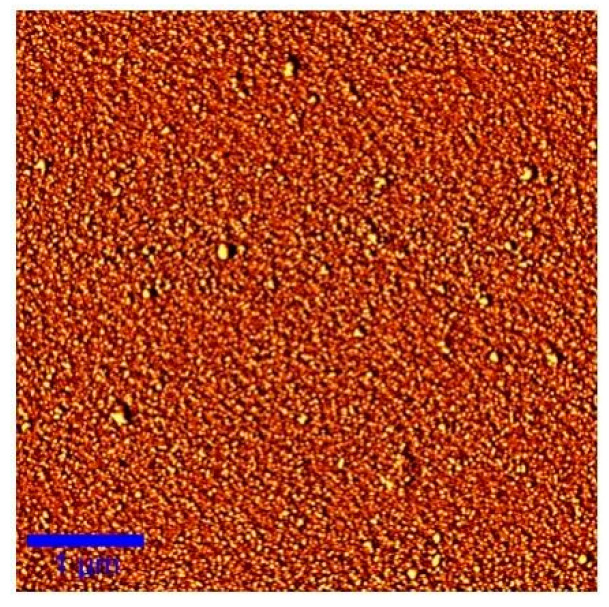
The AFM (Atomic Force Microscope) image of Ge_17.5_As_11_Se_72.5_ thin films.

The AFM image of the Ge_17.5_As_11_Se_72.5_ thin film is demonstrated in Figure 9. The whole image was taken at a scale of 1 × 1 μm. The surface morphology of the films was compact and uniform. The larger particles on the film surface were also found, which were the impurity particles absorbed on the film surface during thermal evaporation. On the whole, the preparation of the thin film was successful without big defects. The uniformity of morphology also indirectly indicated that the microstructure of the material was stable, and its transmission characteristics should be kept at a certain value.

## 4. Conclusions

The series of Ge–As–Se glasses films in which MCN from 2.46 to 2.78 was achieved by this work. Thermal kinetics analysis of in situ measures indicated that the change in crystal structure from an amorphous structure to a melting liquid state. In the crystallization process, the corresponding state-transition temperatures T_x_, T_l_, T_p_ were linear with MCN. Analysis of the results through decomposing the Raman spectra into different structural units showed that the Ge−Ge, As−As, or Se−Se homopolar bonds could be found, and the nonequilibrium minority carriers contributed to the conduction process so that the electrical properties changed greatly. The glasses exhibited homopolar bonds with changing temperature; thus, the lowest thermal loss is promising for applications.

## Data Availability

The authors declare no any limitations in the data availability Statement.
